# Mosquito-Borne Viral Pathogens Detected in Zambia: A Systematic Review

**DOI:** 10.3390/pathogens10081007

**Published:** 2021-08-10

**Authors:** Rachel Milomba Velu, Geoffrey Kwenda, Liyali Libonda, Caroline Cleopatra Chisenga, Bumbangi Nsoni Flavien, Obvious Nchimunya Chilyabanyama, Michelo Simunyandi, Samuel Bosomprah, Nicholus Chintu Sande, Katendi Changula, Walter Muleya, Monicah Mirai Mburu, Benjamin Mubemba, Simbarashe Chitanga, John Tembo, Matthew Bates, Nathan Kapata, Yasuko Orba, Masahiro Kajihara, Ayato Takada, Hirofumi Sawa, Roma Chilengi, Edgar Simulundu

**Affiliations:** 1 Centre for Infectious Disease Research in Zambia, Lusaka P.O. Box 34681, Zambia; Caroline.Chisenga@cidrz.org (C.C.C.); Obvious.Chilya@cidrz.org (O.N.C.); Michelo.Simuyandi@cidrz.org (M.S.); Samuel.Bosomprah@cidrz.org (S.B.); Roma.Chilengi@cidrz.org (R.C.); 2 Department of Disease Control, School of Veterinary Medicine, University of Zambia, Lusaka P.O. Box 32379, Zambia; nicholus.sande@gmail.com (N.C.S.); atakada@czc.hokudai.ac.jp (A.T.); edgar.simulundu@unza.zm (E.S.); 3 Department of Biomedical Sciences, School of Health Sciences, University of Zambia, Lusaka P.O. Box 50110, Zambia; kwenda.geoffrey@unza.zm (G.K.); schitanga@gmail.com (S.C.); 4 Africa Center of Excellence for Infectious Diseases of Humans and Animals, University of Zambia, Lusaka P.O. Box 32379, Zambia; 5 Department of Disease Control and Prevention, School of Medicine and Health Sciences, Eden University, Lusaka P.O. Box 37727, Zambia; libonda.libonda@gmail.com (L.L.); bnflavien@gmail.com (B.N.F.); 6 Department of Biostatistics, School of Public Health, University of Ghana, Accra P.O. Box LG13, Ghana; 7 Department of Paraclinical Studies, School of Veterinary Medicine, University of Zambia, Lusaka P.O. Box 32379, Zambia; katendi.changula@gmail.com; 8 Department of Biomedical Sciences, School of Veterinary Medicine, University of Zambia, Lusaka P.O. Box 32379, Zambia; muleyawalter@gmail.com; 9 Macha Research Trust, Choma P.O. Box 630166, Zambia; monicah.mburu@macharesearch.org; 10 Department of Zoology and Aquatic Sciences, School of Natural Resources, Copperbelt University, Kitwe P.O. Box 21692, Zambia; mubembab85@yahoo.co.uk; 11 School of Veterinary Medicine, University of Namibia, Windhoek Private Bag 13301, Namibia; 12 School of Life Sciences, University of KwaZulu-Natal, Private Bag X54001, Durban 4000, South Africa; 13 HerpeZ Infection Research and Training, University Teaching Hospital, Lusaka Private Bag RW1X Ridgeway, Lusaka P.O. Box 10101, Zambia; john.tembo@gmail.com (J.T.); mbates@lincoln.ac.uk (M.B.); 14 School of Life Sciences, University of Lincoln, Brayford Pool, Lincoln LN6 7TS, UK; 15 Zambia National Public Health Institute, Ministry of Health, Lusaka P.O. Box 30205, Zambia; nkapata@gmail.com; 16 Division of Molecular Pathobiology, International Institute for Zoonosis Control, Hokkaido University, N 20 W10, Kita-ku, Sapporo 001-0020, Japan; orbay@czc.hokudai.ac.jp; 17 Division of Global Epidemiology, International Institute for Zoonosis Control, Hokkaido University, N 20 W10, Kita-ku, Sapporo 001-0020, Japan; kajihara@czc.hokudai.ac.jp; 18 Global Virus Network, 725 W Lombard St., Baltimore, MD 21201, USA

**Keywords:** mosquito-borne, arboviruses, *Togaviridae*, *Flaviviridae*, *Phenuiviridae*, Zambia

## Abstract

Emerging and re-emerging mosquito-borne viral diseases are a threat to global health. This systematic review aimed to investigate the available evidence of mosquito-borne viral pathogens reported in Zambia. A search of literature was conducted in PubMed and Google Scholar for articles published from 1 January 1930 to 30 June 2020 using a combination of keywords. Eight mosquito-borne viruses belonging to three families, *Togaviridae*, *Flaviviridae* and *Phenuiviridae* were reported. Three viruses (Chikungunya virus, Mayaro virus, Mwinilunga virus) were reported among the togaviruses whilst four (dengue virus, West Nile virus, yellow fever virus, Zika virus) were among the flavivirus and only one virus, Rift Valley fever virus, was reported in the *Phenuiviridae* family. The majority of these mosquito-borne viruses were reported in Western and North-Western provinces. *Aedes* and *Culex* species were the main mosquito-borne viral vectors reported. Farming, fishing, movement of people and rain patterns were among factors associated with mosquito-borne viral infection in Zambia. Better diagnostic methods, such as the use of molecular tools, to detect the viruses in potential vectors, humans, and animals, including the recognition of arboviral risk zones and how the viruses circulate, are important for improved surveillance and design of effective prevention and control measures.

## 1. Introduction

Arthropod-borne viruses (arboviruses) are transmitted to susceptible vertebrate hosts by hematophagous, or blood-sucking arthropods such as mosquitoes, sandflies, lice, ticks and fleas [1]. There are an estimated 700 known arboviruses among which about 100 are known to cause infections in humans and animals [2].

Medically important arboviral infections mostly include flaviviral infections such as yellow fever, Zika virus disease, West Nile fever, and dengue fever. In addition, Chikungunya fever caused by Chikungunya virus, an alphavirus has caused many documented outbreaks in Africa, Asia, Europe, the South Pacific and recently the Caribbean region [3,4,5]. The transmission dynamics of these viruses depend on several factors, which may vary from viral genetics to vector competence and ecological interactions between hosts and vectors [3,6].

Mosquitoes belonging to *Aedes* and *Culex* species play an important role in arboviral transmission. For instance, *Aedes aegypti* and *Aedes albopictus* have been incriminated in the transmission of prevalent arboviruses of medical importance. These include Chikungunya virus (CHIKV), dengue virus (DENV), and yellow Fever virus (YFV) [7]. On the other hand, *Culex* mosquitoes are principal vectors of many viruses including Japanese encephalitis virus (JEV), West Nile virus (WNV), and St Louis encephalitis virus (SLEV) among others [8].

The clinical presentation of these viruses is non-specific and sometimes can lead to misdiagnosis as most of them have a very similar clinical presentation. Symptoms can include fever, myalgia, polyarthralgia (migratory polyarthritis), rash, headache, photophobia, hyperemia and in some cases neurological complications such as meningitis, flaccid paralysis, and meningoencephalitis [9,10].

In sub-Saharan Africa, the presence of *Aedes*, *Culex* and *Anopheles* spp., transmitting mosquito-borne viral diseases and malaria are well established [11,12]. The co-circulation of these mosquito-borne viruses with other pathogens causing febrile illnesses such as malaria poses a serious problem of diagnosis and management. This has led to an under-estimation of mosquito-borne viral infections as most of the febrile illnesses are treated as malaria [4,13].

Due to her geographical location and tropical climate, Zambia hosts favourable breeding sites for mosquitoes that transmit malaria and mosquito-borne infections [14] and most of these have been reported for instance, CHIKV was first reported in 1961 [15]. After a long epidemiological silence of about three decades, serological studies have demonstrated evidence of exposure to other mosquito-borne viruses of public health importance such as dengue virus (DENV), yellow fever virus (YFV), Zika virus (ZIKV), Mayaro virus (MAYV) and West Nile virus (WNV) [16].

In light of recent global outbreaks, endemic conditions caused by these mosquito-borne viruses are likely to remain neglected and risk being forgotten. We undertook a systematic review to investigate the available evidence on the distribution of arboviruses and their impact in Zambia, including pre-colonial period.

## 2. Results

### 2.1. Electronic/Manual Searching

The systematic electronic database searching strategy conducted in PubMed and PubMed Central yielded 465 articles. The manual searching employed in Google scholar, Cochrane library and Directory of Open Access Journals [DOAJ] returned 95 articles. Altogether, the total number of retrieved articles was 560, of which 68 were systematic reviews, 33 were editorials and 94 were duplicates, and thus were removed. A total number of 365 articles were subjected to title and abstract screening against the inclusion/exclusion criteria. Of these, 332 articles were excluded because the articles were not conducted in Zambia (*n* = 236) and 96 did not investigate mosquito-borne viruses.

**Figure 1:**
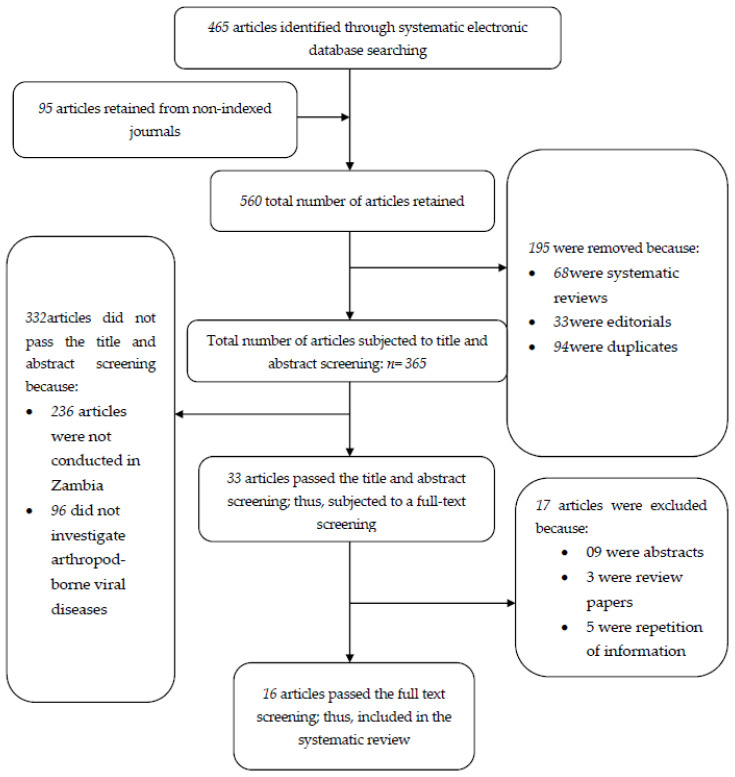
Flow chart diagram describing the literature search process.

The articles which passed the title and abstract screening (*n* = 33) were further subjected to full-text screening against the inclusion criteria by two investigators. After the full-text screening, sixteen (*n* = 16) articles were within the inclusion criteria and were thus included in the present systematic review. The searching process is summarized in Figure 1.

### 2.2. General Study Characteristics

A total of 16 studies were included in this review, and all the 16 studies described the sampling approaches used in selecting the sample. Most of the studies (12) were cross-sectional studies [16,17,18,19,20,21,22,23,24,25,26,27]. Seven research studies were conducted in humans [15,16,22,23,27,28,29] while nine studies were conducted in animals and/or mosquitoes, and one study had both humans and animals [27].

The studies obtained in our search used different study designs as well as sampling strategies to obtain data on the different mosquito-borne pathogens. The study designs employed were cross sectional (12/16), randomized cluster (2/16), retrospective (1/16) and case reports (1/16), with the sampling strategies used included convenience (10/16), two-stage cluster sampling (3/16), multi-stage (1/16), stratified (1/16) and a case report (1/16).

To determine association between prevalence of mosquito-borne viruses and some potential risk factors, bivariate, multivariate and Pearson’s correlation tests were employed in the different studies. More details on study characteristics are provided in Table 1 whilst the prevalence versus risk factors are summarised in Table 2, Table 3 and Table 4.

### 2.3. Geographical Location

Concerning geographical locations, seven of Zambia’s ten provinces reported mosquito-borne viruses. These include Copperbelt, Central, Eastern, Lusaka, North-Western, Southern and Western provinces. However, Western and North-Western provinces had the most common sites for mosquito-borne virus studies as reported in eight and ten studies, respectively.

### 2.4. Mosquito-Borne Viruses

**Figure 2:**
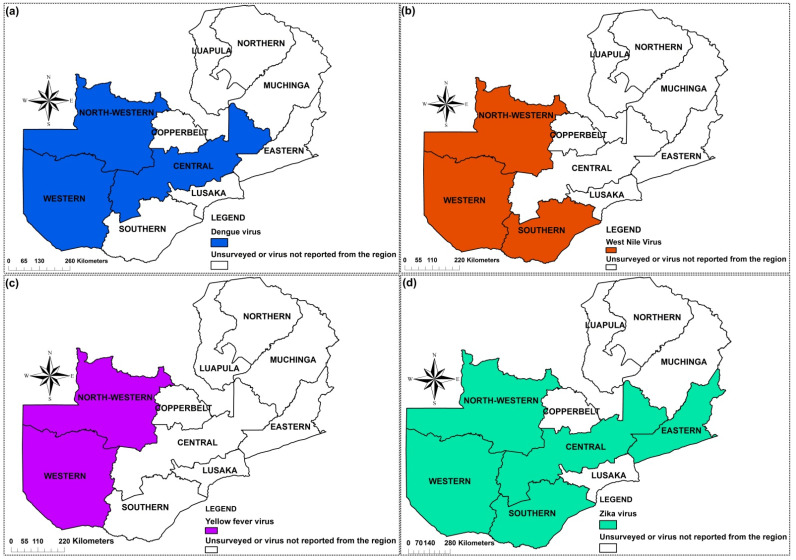
Provinces in Zambia where mosquito-borne pathogens have been reported: Flaviviruses (**a**–**d**).

**Figure 3:**
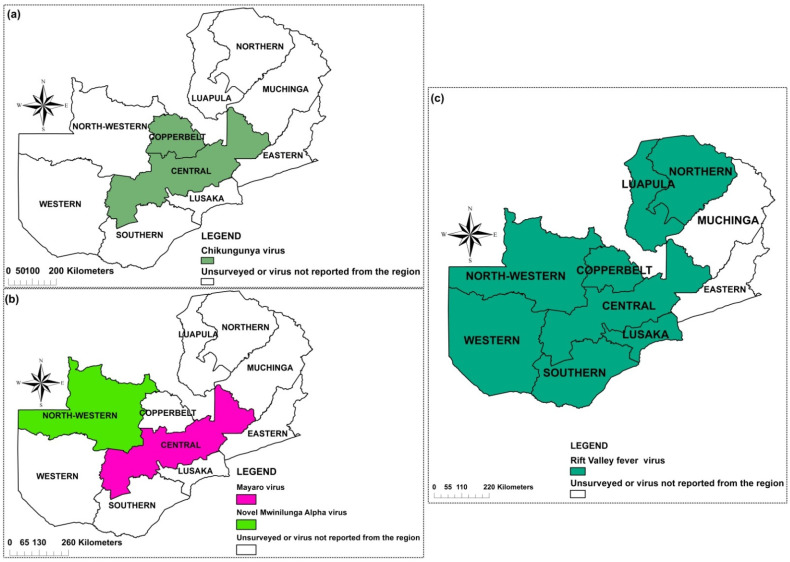
Provinces in Zambia where alphaviruses (**a**,**b**) and Rift Valley fever virus (**c**) have been reported.

Eight mosquito-borne viruses belonging to three families, *Togaviridae*, *Flaviviridae* and *Phenuiviridae* were recorded. Four viruses amid the flavivirus were reported DENV, WNV, YFV, and ZIKV (Figure 2). Among the togaviruses, CHIKV, MAYV and Mwinilunga alphavirus (MWAV) (Figure 3) were reported while among the phenuiviruses, only RVFV was reported (Figure 3).

#### 2.4.1. Dengue Virus

Evidence for the presence of dengue in Zambia was first shown serologically in 1987 [30]. Since then, serological evidence of the virus has been reported in Western [28,31], North-Western [31] and Central Provinces [16] with reported prevalence ranging from 4.1% to 16.8%. Amongst the recognized risk factors for dengue virus infection in a population in Western Province were the age, education and history of travel to Angola [28]. However, there is currently no knowledge of the serotypes circulating in the country [31]. Figure 2a shows the provinces of Zambia where DENV has been reported.

#### 2.4.2. West Nile Virus

Serological evidence of WNV in Zambia was first reported in 2015, with a prevalence of 10.3% [29]. This is the only study and report of WNV in humans in the country. Other reports of WNV in Zambia were in *Culex* mosquitoes [21] at a prevalence of 6.7% and in farmed crocodiles [17]. Genetic characterization studies have revealed the presence of lineages 1a [17] and 2 [21] in the country. The identification of the virus in mosquitoes shows that the virus could be circulating within the communities, thus heightens the need for proper understanding of disease epidemiology in those communities. Figure 2b shows the provinces in Zambia where WNV has been reported.

#### 2.4.3. Yellow Fever Virus

The first serological survey of YFV could have occurred in Zambia in the then Northern Rhodesia, between 1937 and 1943 [32]. Since then, subsequent studies have been conducted in the country. In 1950, over 7% of more than 3000 blood samples from the population of the Zambezi district in Kaonde-Lunda Province now called North-Western Province provided positive results to YFV [33]. Between 1951 and 1953, many seroprevalence studies were conducted in the Zambezi River basin and the prevalence rates were ranging from 0.4% to 11% depending on the age group [34]. After almost six decades, a WHO Yellow Fever Technical Working Group classified the provinces along the Zambezi River basin, North-Western and Western as yellow fever (YF) low-risk regions [35].

A seroprevalence survey was conducted in two provinces (Western and North-Western) to ascertain the potential risk of YF infection. The findings revealed a prevalence of 0.3% for long-term infection and 0.2% for recent YF infection in the two provinces, respectively [22]. The distribution of YFV in Zambia is shown in Figure 2c.

#### 2.4.4. Zika Virus

Two studies conducted on humans on ZIKV were identified during the review. The first study was conducted in 2015 to determine the prevalence and the risk factors for Zika virus infection in the Western and North-western provinces of Zambia. Out of a total of 3625, 6.1% of participants had Zika virus antibodies. Factors such as age, indoor residual spray, type of roof and visiting Angola were associated with Zika virus infection in bivariate analyses [36]. In the second study, Chisenga et al. reported a ZIKV seroprevalence of 10.8% (23/214) from serum samples collected in Central Province [16]. In 2019, 96 Non-human primates (African green monkeys and baboons) from Southern and Eastern provinces were tested for ZIKV using plaque reduction neutralization test and RT-PCR. The findings revealed that 34.4% of their sera had neutralizing antibodies against ZIKV whereas the ZIKV genomic RNA was not detected using RT-PCR [18]. The distribution of ZIKV across the country is summarised in Figure 2d.

#### 2.4.5. Chikungunya Virus

CHIKV was first reported in 1961 in Luanshya, Copperbelt Province during an outbreak where similar cases of febrile conditions of sudden onset with headache, photophobia, pain in the joints and muscles, sore throat, occasional cough, and a maculo-erythematous rash appearing between the second and sixth days were observed. Three samples out of 7 tested positive for CHIKV IgM antibodies [15]. Since then, CHIKV was not reported in the country until close to six decades later. Blood collected during a mass-cholera vaccine trial in 2016 was used to screen for arboviruses in Central Province. Two hundred and fourteen serum samples were available for testing IgG antibodies against CHIKV, DENV, MAYV and ZIKV arboviruses, out of which 79 (36.9%; 95% CI 30.5–43.8) were seropositive for CHIKV [16]. Figure 3a represents provinces were CHIKV has been reported.

#### 2.4.6. Mayaro Virus

MAYV, an *Alphavirus*, member of the *Togaviridae* family together with CHIKV, has been limited to Central and South America [37]. Very few studies have been reported in Africa. In Zambia, one study, conducted in Central Province (Figure 3b) and published in 2020 is the only record of possible MAYV presence in the country. Its prevalence was estimated to be 19.6% (42/214) [16].

#### 2.4.7. Mwinilunga Alphavirus

Mwinilunga alphavirus (MWAV) is a newly discovered virus. It was first reported from a single *Culex quinquefasciatus* mosquito pool in Zambia in 2018, and its genome has since been sequenced. From the 9699 mosquitoes collected in North-Western Province (Figure 3b), a 0.2% prevalence of the virus was obtained [20]. However, studies in humans have not been conducted, and its pathogenicity in human or other vertebrates is not well established.

#### 2.4.8. Rift Valley Fever Virus

Rift Valley fever virus (RVFV) was first reported in 1985 after a seroprevalence study conducted in 440 cattle and sheep in Central Province using a complement fixation test [38]. In 1992, serum samples from cattle and sheep, sampled from Lusaka, Copperbelt and Central provinces showed evidence of epizootic Rift Valley fever (RVF) in Zambia [26]. Five years later, Samui et al. reported a positivity rate of 10.5% to RVFV in 27 herds of cattle [25].

Using a recently developed diagnostic tool on a recombinant nucleocapsid protein (rNP)-based indirect immunofluorescent antibody assay (IFA), it was found that the seroprevalence of RVF varied between 6.0% to 21.4% among cattle herds in Central, Southern and Western provinces [19].

A study conducted on humans in 1987 showed that 5 out of 53 (9.4%) workers at an abattoir dealing with cattle in Lusaka were seropositive for RVF, but none of the workers at the abattoirs dealing with pigs was shown to be positive [27]. Figure 3c shows the locations where RVF has been reported in the country.

### 2.5. Factors Associated with Mosquito-Borne Viruses’ Distribution in Zambia

Several factors have been associated with mosquito-borne viral infections globally and in Zambia. Anthropological activities, such as deforestation, land use patterns, demographic density, global trade, and global warming (climate change) have all successfully contributed to the emergence and re-emergence of these viruses [39]. Human activities including farming and fishing have been incriminated in CHIKV infection in the Copperbelt and Central Provinces [15,16].

The movement of people to neighbouring countries is most likely contributing to arboviral activity in Zambia. For instance, visiting Angola was associated with ZIKV and DENV infections [28,36], whereas travelling to the Democratic Republic of Congo (DRC) and South Africa was significantly related to YFV infection [22]

The transmission dynamic of mosquito-borne viruses has also been associated with climate conditions. Most of RVF epidemics have been reported during the rainy seasons [25,26].

### 2.6. Mosquito Species from Which Potential Zoonotic Arboviruses Have Been Detected in Zambia

After the first record of mosquitoes belonging to *Aedes* species in 1950 [33], information has been scarce regarding the distribution and species composition of mosquito vectors in Zambia. An entomological investigation conducted in North-Western and Northern provinces to identify yellow fever vectors showed that *Aedes* (*Stegomyia*) *aegypti* (*Ae. aegypti*) and *Aedes* (*Stegomyia*) *africanus*, the two main vectors of YFV, were present in the study sites in low densities [24]. Other mosquito species identified included *Aedes* (*Aedimorphus*) *mutilus*; *Aedes* (*Aedimorphus*) *minutus*, *Aedes* (*Finlaya*) *wellmani*, *Culex* species (Cx. *quinquefasciatus*) and *Mansonia* species (*Mansonia africanus*).

Mosquito-borne viruses have been detected in mosquitoes on only two occasions in Zambia. The first occasion was in 2017, where the WNV lineage 2 strain was isolated in a pool of *Culex* mosquitoes collected from Western Province [21]. Furthermore, a novel alphavirus, tentatively named Mwinilunga alphavirus was identified from *Culex quinquefasciatus* mosquitoes in 2018 [20]. This discovery highlights the necessity of conducting robust entomological surveillance in the country for an improved understanding of mosquito-borne viral transmission dynamics in Zambia.

## 3. Discussion

The evidence gathered in this review indicated that a considerable array of mosquito-borne viruses have been detected in Zambia. Although no epidemics have been reported in the country, there is evidence of an on-going mosquito-borne viral activity, mostly in North-Western, Western and Southern provinces [17,20,21,22,25,31]. It was interesting to note that Muchinga Province did not report any form of mosquito-borne viral activity, seroprevalence evidence or virus isolation. Whichever the case, it is cardinal to do studies in this part of the country to ensure an understanding of the prevailing situation.

Although most of the research studies did not elaborately highlight climatic conditions to influence the distribution of mosquitoes, Masaninga et al. (2014) observed climatic factors such as precipitations and temperature to somewhat influence their distribution. Further, it was noted that North-Western, Western, and Southern provinces host favourable breeding sites for mosquitoes due to their humid conditions, increasing the risk of mosquito-borne viruses [24]. This observation is in agreement with studies on DENV infection in Africa which hypothesised climatological patterns to favour vector development and longevity as humid warm tropical regions promote egg conservation and proliferation [40].

Following the analysis of the rainfall patterns, it was shown that provinces which receive on average 900 mm or higher than 1000 mm of rainfall are predisposed to high incidences of mosquito-borne diseases due to an increase in vector diversity and activities [41]. RVF epidemics reported in 1992 and 1997 by Davies et al. (1992) and Samui et al. (1997), respectively, were related to high precipitation. Similar observations were noted in Kenya where RVF epizootics were associated with relatively high levels of rainfall [42,43].

Of note, human activities such as deforestation, trade and movement of people and animals have been associated with the introduction of mosquito-borne viruses in previously non-endemic regions [44,45]. Human movement has also been linked to ZIKV, DENV and YFV seropositivity in Zambia [28,46]. This is also plausible because the country shares borders with countries where major outbreaks of these arboviruses have been previously reported [44,47]. It is also reasonable that livestock trade between Zambia and her neighbouring countries likely promulgates the distribution of mosquito-borne viruses [41].

However, although different methods were used in the studies included with regards to detecting mosquito-borne viruses and thus introducing heterogeneity, the review largely revealed information on circulating mosquito-borne viruses and their role in the transmission dynamics of mosquito-borne viral infections; therefore, conducting more entomological studies to investigate the vector population and associated infections is crucial to control mosquito-borne viral diseases.

## 4. Materials and Methods

### 4.1. Search Strategy

Using PRISMA guidelines for systematic review, we searched for information related to mosquito-borne viral pathogens found in Zambia on PubMed and PubMed Central electronic databases from 1 January 1930 to 30 June 2020. A manual search was done on Google scholar, Cochrane library and Directory of Open Access Journals [DOAJ].

The search strategy involved a combination of keywords (“Northern Rhodesia” OR “Republic of Zambia”) AND (“Mosquito-borne virus” OR “Arthropod-borne virus” OR “Arbovirus” OR “Dengue virus” OR “Yellow fever virus” OR “Chikungunya virus” OR “West-Nile virus” OR “Rift Valley fever virus” OR “Zika virus” OR “Mayaro virus” OR “flavivirus” OR “phlebovirus” OR “alphavirus” OR “bunyavirus”) AND (“Epidemiology” OR “Prevalence” OR “Distribution”).

The articles retained were populated in RefWorks (2020) database manager, and duplicates were automatically removed. Titles and abstracts of retained articles were subjected to the inclusion and exclusion criteria.

### 4.2. Inclusion Criteria

All primary studies which focused on the occurrence and distribution of mosquito-borne viral diseases in Zambia from 1 January 1930 to 30 June December 2020 published in peer-reviewed journals, in either English or French languages, were included. Demographic health surveys indicating the burden of mosquito-borne viral diseases were also considered.

### 4.3. Exclusion Criteria

Studies published in languages other than English or French, or lacking extractable data or not explicit in methodology, and systematic review papers were excluded. Abstracts without full manuscript texts were also excluded.

### 4.4. Outcome Measures

The primary outcome was the distribution of mosquito-borne viral diseases. The secondary outcomes were the risk factors influencing their distribution.

### 4.5. Data Extraction

Potentially eligible articles were selected and screened using their title and abstract by two independent reviewers (RV and LL). The articles were divided into 2 subgroups “included” and “excluded” using the set inclusion and exclusion criteria. The final inclusion was done by analysing the full texts of the included articles. When necessary, any disagreements were resolved by arbitration of the third reviewer (BF). Data was extracted using an extraction tool from the Joana Briggs Institute Reviewers Manual (2018) for prevalence studies. Information regarding the authors, location, study design, characteristics of the sampled population, diagnostic tests used, and specific results was extracted and entered into an Excel sheet.

### 4.6. Distribution Mapping

The data extracted was used to create a map of the distribution and/or occurrence of mosquito-borne viruses across the country using ArcGIS version 10.3 (Figure 2 and Figure 3).

### 4.7. Data Synthesis

A narrative summary of included studies was done by pooling the raw data with an emphasis on reporting their characteristics along with data extracted relevant to the review outcomes. Analysis of quantitative studies was done based on the heterogeneity of the included studies.

## 5. Conclusions

The findings of this review demonstrate that mosquito-borne viruses constitute a public health threat to the country. Despite this threat, very few studies have been conducted to understand the virus, vector and reservoir host interactions and dynamics. Though no epidemic has been reported as yet, favourable ecological factors noted in the country may lead to a rise in cases of mosquito-borne viral infections. Accurate information regarding the epidemiology and ecology of mosquito-borne viruses is of critical importance for implementing suitable surveillance strategies, prophylactic treatments, travel recommendations and clinical therapies. Furthermore, better detection methods, such as molecular tools, to detect the viruses in potential vectors, humans and animals, including the recognition of arboviral zones and how the viruses circulate, are important for improved surveillance and better appreciation of the impact of these viruses on animals and humans.

## Figures and Tables

**Table 1:** General study characteristics.

Author and Year	Study Design	Sampling Strategy	Eligibility Criteria Described	*n* Pre/Post	Powered Sample	Confounders Tested	Data Collection Tools-Pilot
Chisenga et al. (2020)	Cross-sectional study	Convenience sample	Adults aged 18 years and above	173/214	* NM	Sex, age, occupation, blood group	* NM
Simulundu et al. (2020)	Cross-sectional study	Convenience sample	* NM	11	* NM	* NM	* NM
Wastika et al. (2019)	Cross-sectional study	Convenience sample	* NM	96	* NM	* NM	* NM
Ngonda et al. (2018)	Cross-sectional study	Convenience sample	** N/A	942	** N/A	** N/A	** N/A
Torii et al. (2018)	Cross-sectional study	Convenience sample	** N/A	9699	** N/A	** N/A	** N/A
Orba et al. (2017)	Cross-sectional study	Convenience sample	** N/A	9439	** N/A	** N/A	** N/A
Babaniyi et al. (2015)	Cross-sectional study	Multi-stage sampling technique	Persons aged nine months or older	* 3600/3625	Yes	Age, sex, education, indoor residual spraying, visiting Angola, type of roof	* NM
Mweene-Ndumba et al. (2015)	Randomized cluster design	Two-stage cluster sampling technique	Persons aged nine months or older	3612/3625	Yes	Age, sex, occupation, education, roof type and use of mosquito insecticide-treated nets and indoor residual spray	* NM
Babaniyi et al. (2015)	Cross-sectional study	Two-stage cluster sampling technique	Persons aged nine months or older	3625/3579	Yes	Age, sex, education, material for roof, material for wall, travelling outside Zambia	* NM
Masaninga et al. (2014)	Cross-sectional study	Convenience sample	* NM	* 1465/1465	* NM	* NM	* NM
Mazaba-Liwewe et al. (2014)	Randomized cluster design	Two-stage cluster sampling technique using probability proportional to size.	Persons aged nine months or older	3612/3624	Yes	Sex, age, education, roof type, occupation, visiting Angola	* NM
Samui et al. (1997)	Cross-sectional study	Convenience sample	** N/A	1421	** N/A	**N/A	* NM
Davies et al. (1992)	Cross-sectional study	Convenience sample	** N/A	387	** N/A	** N/A	** N/A
Morita C. (1988)	Cross-sectional study	Convenience sample	Abattoir personnel	407		Sex, age, duration of employment	* NM
Hussein et al. (1985)	Retrospective study	Stratified sample	* NM	440	* NM	Age	* NM
Rodger (1961)	Case report	** N/A	** N/A	13	** N/A	** N/A	** N/A

* NM = Not Mentioned, ** N/A = Not Applicable, * 3600/3625 = ZIKV, * 1465/1465 = Only 53 samples tested for YFV.

**Table 2:** Apparent prevalence in humans and risk factors reported in the reviewed studies.

Author	Population	Sample Size	Laboratory Techniques	Arbovirus Prevalence	Risk Factors		
					Age	Occupation	Movement of People
Chisenga et al. (2020)	Adults	N = 214	ELISA	36.9% (CHIKV); 10.8% (ZIKV); 16.8% (DENV); 19.6% (MAYV)	* SS (OR = ? 95% CI: 9.6–37.3, for ZIKV)	* SS (OR = ? 95% CI: 34.5–51, for CHIKV)	** NE
Mazaba-Liwewe et al. (2014)	Children (9 months and above) and Adults	N = 3612	ELISA	7.1% (DENV)	* SS (OR = 1.66; 95% CI: 1.1–2.5)	** NE	* SS (OR = 2.11; 95% CI: 1.41–3.15)
Mweene-Ndumba et al. (2015)	Children and Adults	N = 3625	ELISA	10.3% (WNV)	* SS (OR = 1.49; 95% CI: 1.14–1.94)	* SS (OR = 0.80; 95% CI: 0.64–0.99)	* SS (OR = 1.40; 95% CI: 1.09–1.81)
Babaniyi et al. (2015)	Children and Adults	N = 3625	ELISA and PRNT	0.3% (YFV)	** NE	** NE	* SS (P < 0.001)
Babaniyi et al. (2015)	Children andAdults	N = 3579	ELISA	6.1% (ZIKV)	* SS (AOR = 0.36; 95% CI: 0.18–0.72)	** NE	* SS (AOR = 1.42; 95% CI: 1.06–1.90)
Morita C. (1988)	Adults	N = 102	Cell culture and indirect IFA test	9.4% (RVFV)	*** E (** SS?)	*** E (** SS?)	** NE
Rodger (1961)	Children (15 years) and Adults	N = 13	CFT and Agglutination inhibition test	0.4% (CHIKV)	** NE	*** E (SS?)	** NE

* SS: Statistically Significant; ** SS?: Not sure if Statistically Significant, ** NE: Not Examined; *** E = Examined; IFA: Immunofluorescent antibody; PRNT: Plaque Reduction Neutralization Testing, CFT: Complement Fixation Test.

**Table 3:** Apparent prevalence in animals and mosquitoes reported in the reviewed studies.

Author	Population	Sample Size	Laboratory Techniques	Arbovirus Prevalence
Simulundu et al. (2020)	Crocodiles	N = 11	RT-PCR	** NE
Wastika et al. (2019)	Non-human primates	N = 96	PRNT and RT-PCR	34.4% (ZIKV)
Orba et al. (2018)	Mosquitoes	N = 9439	RT-PCR, virus isolation, NGS	6.7% (WNV)
Saasa et al. (2018)	Cattle	N = 942	Indirect IFA assay	** NE (RVF)
Masaninga et al. (2014)	Mosquitoes	* N = 1466	PCR	0% (YFV)
Torii et al. (2018)	Mosquitoes	N = 9699	RT-PCR	0.2% (MWAV)
Samui et al. (1997)	Cattle	N = 1421	IFA	10.5% (RVFV)
Davies et al. (1992)	Cattle and Sheep	N = 387	IFA and VSNT	80% (RVFV)
Morita C. (1988)	Cattle	N = 212	Cell culture and indirect IFA test	0% (RVFV)
Hussein et al. (1985)	Cattle and Sheep	N = 440	CFT	47.4% and 2.5% (RVF)

** NE: Not Examined; IFA: Immunofluorescent antibody; PRNT: Plaque Reduction Neutralization Testing, CFT: Complement Fixation Test, * N = 1466: only 53 were tested; VSNT: Virus Serum Neutralization Test.

**Table 4:** Summary of reported apparent prevalence in the sampled population.

Sampled Population	Arbovirus							
	CHIKV	ZIKV	DENV	MAYV	MWAV	RVFV	WNV	YFV
Humans	0.5–100% (50.3%)	1.8–10.8% (6.3%)	4.1–16.8% (10.5%)	19.6%	____	____	10.3%	0.3%
Mosquitoes	____	____	____	____	0.2%	____	6.7%	____
Cattle	____	____	____	____	____	10.5–80% (45.3%)	____	____
Sheep	____	____	____	____	____	2.5–80% (41.3%)	____	____
African green monkeys and baboons	____	34.4%	____	____	____	____	____	____

CHIKV: Chikungunya Virus, ZIKV: Zika Virus, DENV: Dengue Virus, MAYV: Mayaro Virus, MWAV: Mwinilunga alphavirus, RVF: Rift Valley Fever Virus, WNV: West-Nile Virus, YFV: Yellow Fever Virus.

## Data Availability

Data is contained within the article.
